# Lone workers attitudes towards their health: views of Ontario truck drivers and their managers

**DOI:** 10.1186/1756-0500-7-297

**Published:** 2014-05-14

**Authors:** Beatrice McDonough, Michelle Howard, Ricardo Angeles, Lisa Dolovich, Francine Marzanek-Lefebvre, John J Riva, Stephanie Laryea

**Affiliations:** 1 Department of Family Medicine, McMaster University, McMaster Innovation Park, Suite 201A, 175 Longwood Road South, Hamilton, ON L8P 0A1, Canada; 2 Healthy Living Division, City of Hamilton Public Health Services, 1447 Upper Ottawa, Unit 10, Hamilton, ON L8W 3J6, Canada; 3 Centre for Evaluation of Medicine, 105 Main Street East, Level P1, Hamilton, ON L8N 1G6, Canada; 4 Pan-American Health Organization/World Health Organization, Paramaribo, Suriname

**Keywords:** Truck drivers, Health and wellness, Chronic disease risk factors, Lifestyle, Workplace health

## Abstract

**Background:**

Truck driving is the second most common occupation among Canadian men. Transportation of goods via roads is of crucial importance for the Canadian economy. The industry is responsible annually for $17 billion in GDP and is projected to increase by 28% over the next 10 years. Recruitment is an issue with 20% of drivers projected to retire or leave the profession in the next 10 years. Despite the reliance on transport truck drivers for the delivery of goods which affects Canada’s economy and daily living of residents, little is known about the health care needs of this large cohort of primarily male lone workers from a drivers’ perspective. Transport truck drivers are independent workers whose non traditional workplace is their tractor, the truck stops and the journey on the road.

The objective of this study was to obtain a contextually informed description of lifestyle issues, health and disease risk factors experienced by drivers and perceived by their managers in the truck driving occupation.

**Methods:**

Using a grounded theory approach, 4 focus groups were conducted with drivers (n = 16) and managers (n = 10) from two trucking companies in Southwestern Ontario to identify the lived experience of the drivers as it relates to preventable risks to health and wellness. A semi structured guided interview was used to explore the lifestyle context of transport truck driving and organizational aspects of the occupation (workplace culture, working conditions and health and wellness promotion).

**Results:**

The predominant themes described stress, workplace, communication, lifestyle, driving culture, family, and fatigue concerns. In terms of the transportation work environment, drivers and managers were aware of the profession’s potential to foster lifestyle related chronic diseases but described challenges in making the profession more amenable to a healthy lifestyle.

**Conclusions:**

Workplace environmental determinants are significant in shaping health behaviours. Chronic disease health risks were the main health concerns identified. Health risks were exacerbated by working conditions (job demands, work hours, financial pressure and the sedentary nature of the job). Workplace health strategies will need to take into account the unique challenges of the occupation.

## Background

The transport trucking industry plays a major role in the Canadian economy with more than half of all imported and exported products being transported by truck. Over 300,000 workers (nearly 1% of the Canadian population) and over 1.5 per cent of the labour force are employed as for-hire and private truck drivers in Canada, with Ontario employing the largest number of any province [1,2]. Truck drivers represent the second most prevalent occupation for men in Canada [3]. Truck drivers have challenging lifestyles because of the nature of their job that is characterized by long trips, the highly competitive nature of the sector and an income structure that encourages increased work hours for higher earnings. Long hours of sedentary work has been associated with harmful effects on health, and is often associated with unhealthy lifestyles, such as lack of physical activity, lack of sleep and unhealthy eating habits [4-8]. This lifestyle contributes to one of the highest proportions of lost work time for health reasons in the labour market, at 3.7% for male drivers (compared to 2.6% for all workers) [9]. In Ontario, transport occupations have the highest number of lost-time claims [9]. Obesity, physical inactivity, unhealthy eating, high blood pressure, diabetes, sleep apnea and tobacco use are common among drivers, which may impact costs, productivity and safety in the industry [10-12]. Studies of United States drivers have shown increased risks for heart attacks, hypertension, ulcers, cancer and premature mortality [13-16]. However, there are no reports on the extent of health and lifestyle issues among Canadian truck drivers. Data on the health and risk factors in this population is needed to guide the development of feasible workplace programs, policies and strategies for health improvement in Canada. Conventional office-based health, wellness or chronic disease management strategies may not apply well to this occupation of lone independent workers whose working environment is on the road. Given the very strong interaction between employment conditions and cardiovascular health [15,16], development of innovative cardiovascular disease prevention and management strategies are required to impact this unique population. The objective of our study was to create and administer a relevant health and wellness survey for use in the transport driver population. We conducted a qualitative analysis to obtain a contextually informed description of lifestyle issues and disease risk factors experienced by workers and perceived by their managers in the occupation.

## Methods

### Approach

In 2010, focus groups (FG) were conducted using a grounded theory approach to understand the reality of the day to day life of truck drivers working in the transportation sector in relation to their lifestyle issues, working environment and disease risk [17,18]. Self reported data collected from a semi -structured focus group interview guide resulted in generation of categorical themes through content analysis of the real-life narratives.

### Participant recruitment

Through initial discussions with several local transport companies in and near Hamilton, Canada, two companies agreed to assist the research team in reaching the identified population. The transport company occupational health and safety representatives distributed the study invitation to recruit FG participants. Sixteen truck drivers responded. FGs took place at the company office. Ten management staff were also recruited, two from one company and eight from another company, to participate in separate FGs. The Hamilton Health Sciences/Faculty of Health Sciences McMaster University Research Ethics Board approved our study.

### Interview guide

A semi-structured interview guide was developed based on knowledge of chronic disease risks relating to lifestyle and workplace health and input from management of the two collaborating companies. The interview guide explored transport truck driving in the context of self reported modifiable chronic disease risk factors (e.g. physical inactivity, fatigue, stress, unhealthy eating, unhealthy weight, and tobacco, substance and alcohol use). In addition, organizational aspects such as workplace culture, working environment and workplace health and wellness approaches for the trucking industry were explored. The interview flow began with broad questions regarding perceptions of health, and then progressed to specific questions concerning the nature of their employment. The final questions queried participant interest in the future development of appropriate workplace and chronic disease prevention interventions.

Facilitators were members of the research team. Written informed consent was obtained from participants. To facilitate open discussion and reduce the likelihood of discomfort in discussion of employment topics, manager and driver FGs were separated. It was also stressed to drivers that choice of participation would not affect employment status. Focus groups were audio-recorded and transcribed verbatim. Participants completed a post-interview form that captured demographic information on age, income, education, driver status (company, independent, long or short haul), language, marital status and length of time employed as a driver or manager.

### Analysis

A data-driven content analysis approach was used to analyse the transcripts [17,18]. Transcripts were divided among the research team (BM, MH, RA, FM-L, JR) such that two team members read each transcript independently. Coding rules were created through team discussion using examples from the data. After three iterations of review, common themes emerged that were used to build a coding scheme. When no new codes emerged and consensus was achieved, the transcripts were divided among pairs of team members and coded independently using the scheme to capture the content within each node. The codes were used to develop major themes and sub-themes that encompassed the lifestyle and health issues described for that occupation.

We decided, ‘a priori’, only to present sub-themes that were reported by a minimum of 2 respondents. Our selection of quotations for presentation was guided by consensus between the team members. Triangulation was achieved by comparisons of coding between the paired team members and among the full group.

## Results

Two focus groups (FG) with 8 drivers each, one FG with 8 management personnel, and one with 2 management personnel were conducted during the fall of 2010. Table 1 describes the demographic characteristics of the FG participants.

**Table 1:** Demographic characteristics of respondents who provided comments

**Demographic variables**	**Truck drivers (N = 16)**	**Managers (N = 10)**
Age (mean)	47 years	36 years
Range	23- 66	27-50
Gender		
Male	15	10
Female	1	0
Years employed in industry		
(mean)	19 years	13 years
Range	18 months to 45 years	4 - 32 years
Employment status (number)		
Independent owner	5	0
Company employee	11	10
Perception of their health		
Excellent	0	0
Very Good	7	1
Good	4	7
Fair	1	2
Poor	1	0
No response	3	

The average age of the driver participants was 47 years with a range of 23 to 66 years. They had been working on average for 19 years in the transport sector with a range from 18 months to 45 years. The managers were somewhat younger than the drivers and had typically been working a relatively shorter time in the industry (mean 13 years).

The frequency of comments related to the themes and sub-themes by drivers and management staff are shown in Table 2. Analysis yielded the following four thematic categories: (1) Stress, (2) Workplace and Communication, (3) Lifestyle, Culture and Family, and (4) Fatigue.

**Table 2:** Coding tree for respondents who provided comments

**Themes (4)**	**Sub-themes**	**Number of endorsements**	
		**Driver N = 16**	**Management N = 10**
Stress	Traffic and route (other drivers, last minute loads, short haul versus long haul, weather, border issues, inspection stations)	14	6
	Truckers interaction with Industry, Government, and Public (respect issue, fines, company communication, customer regulations, changing government regulations)	21	7
	Finances (wage issues, payment variations, fines, benefits, regulations)	13	8
Workplace and communication	Training and Regulations (safety, mandated sleep regulation, lack of accredited training schools, cannot leave truck)	12	4
	Relationships with customer (waiting times, lack of respect, regulations)	12	7
	Relationships with employer (just in time delivery, regulations, communication, get product there no matter what)	13	27
	Infrastructure and environment (truck break downs, pollution, truck repairs)	1	1
Lifestyle and Family Dynamic (resulting from 1 and 2)	Nutrition (accessibility, availability, affordability, time, parking issues, lack of healthy choices)	25	19
	Physical activity (no time, too tired, long working hours, sedentary job, scheduling)	19	7
	Culture among drivers (independent, take pride in job, hard to change, generational, like family)	19	10
	Family concerns (late for family events, missed events, guilt)	15	1
	Primary health care conditions (smoking, weight, alcohol, blood pressure, diabetes, asthma, arthritis, musculoskeletal)	16	10
Fatigue	Work hours scheduling (irregular hours, work on demand, long hours)	5	3
	Sleep policy/regulations	5	1
	Alertness, boredom (mental fatigue, need to be ‘on guard’)	2	2

### Stress

Daily stress on the job relating to traffic conditions and route concerns, finances, regulations, rules and workplace communication were identified as the primary themes of importance by the drivers. All drivers identified stress and its components more frequently as a factor in their day- to- day work than did managers. Stressful experiences encountered regularly for drivers included long waiting times for loading and unloading, pressure to meet deadlines, traffic congestion, last minute employer requests for out-of-town trips at rush hour, border crossings, government roadside inspection stations, and mechanical breakdowns. Most drivers also perceived a lack of respect from all sectors: industry, government and the public. Some drivers rationalized stress saying. “There’s no use getting upset about it, because it (last minute requests, disrespect) happens every day and how many times a day right?”

FG participants consistently expressed financial concerns as a source of stress, noting the increasing costs of their work in terms of fuel, traffic tickets for illegal parking in many cases to access food, truck inspection regulation fines, and truck repairs for those who are independent contract drivers. There was also a perceived lack of appropriate increases in earnings, leading to added stress in being able to adequately provide for their families. A driver indicated the lack of consistency of financial compensation within the transport industry. His perception was also that driver remuneration was much less than the perceived comparable job of construction worker.

Many drivers also described the differences in stress between long-haul versus short- haul drivers, and company versus independent drivers. Long-haul drivers are away from home for days to weeks, and are subject to extra complexity due to border-crossing. Short-haul drivers tend to work more in areas of high traffic congestion, are pressured to be on time while often taking extra and last minute loads in a workday, and have problems with access to healthy food. The company drivers are provided with consistent salary, company trucks and extended health benefits. Interestingly, many drivers were unaware of their company benefits. Independent drivers are paid by the trip, load or mile and need to manage their own benefits and truck infrastructure expenses leading to less certainty in income at the end of the month.

### Workplace and communication

Across the FGs, the drivers perceived that workplace organization and regulations put drivers at risk for health and safety problems. Lack of appropriate training before drivers begin working in the field was identified as an initial gap. Many drivers noted the deficiency of accredited truck driving schools which can lead to inexperienced drivers being poorly prepared to drive a truck loaded with product. The managers voiced frustration with trying to schedule workplace training with the varied schedules/ shifts that drivers worked and the desire of the drivers to be with their family after a 12–14 hour day on the road.

In addition, the drivers reported that regulations and customer restrictions added to the feelings of stress and disrespect. For example, due to customer rules and safety policies, it is common for drivers to be confined while waiting for the unloading of their truck. One manager noted that “if they’re allowed in the building (to load or unload their product) they have to stay in a caged area ....they are confined”.

The managers were aware of and sensitive to their role in terms of providing a source of general and health-specific information and education for the drivers but expressed frustration with how to move forward in this area. Examples of health-specific information provided were: attempts at newsletters, occupational health and safety meetings and an open door policy. However, managers were unaware of what workplace health promotion resources were available, how they could connect with drivers given the working hours and independent nature of the work and cautioned that a health and wellness approach must make sense from a business perspective. Drivers felt that companies were not interested in health education from a workplace intervention point of view, and that it was up to the driver to educate himself.

“Work is pulled by our customer” summarizes the issue of the perceived lack of power expressed by both managers and drivers in the relationships with their customers. The competitive for-profit nature of the business was felt to be a challenge for workplace communication and provision of time for workplace health interventions. Responding to customer needs takes priority over and challenges the ability to make truck driving a profession that allows for healthy lifestyles. The managers expressed the tension of making changes for their workers without compromising the competitiveness of the business.

### Lifestyle, culture and family

#### Nutrition

The workplace demands were described as contributing directly to unhealthy lifestyle behaviours. There was awareness that truck stop food options encouraged diets low in fruit and vegetables and high in saturated fat, calories and salt. As drivers lamented “Parking is an issue so you can’t get proper food. You go to a truck stop you know…and look at the menu. Everything is like ‘drips with grease’. … and if they do offer fresh fruit… its way over-priced”.

Drivers were also aware that poor eating habits and the sedentary nature of their job lead to unhealthy weight gain but could not see how to change the situation.

The nutrition concerns identified by the majority of the drivers and managers related to accessibility, availability, affordability, and the time required to purchase food. Managers recognised the problems and attempted to identify strategies to address the issue however were unable to come up with alternatives that did not cut into the ‘bottom-line’.

#### Physical activity

Lack of physical activity was mentioned more by drivers than management as an area of concern. Drivers recognized that they needed physical activity but commented “it’s hard to have a healthy lifestyle when you’re working 16 hours a day…” and “sitting sometimes 6–10 hours driving or waiting to load or unload”. Drivers who drove flatbed trucks felt that they accumulated more exercise since they were tarping, tightening chains, and moving up and down from the trailer. They felt they obtained at least an hour of hard physical work a day.

#### Driving culture

Both drivers and managers commented on the unique culture among drivers. The isolated, mobile nature of the work meant that drivers must perform most physical tasks in isolation without assistance or social reinforcement for safety procedures. Productivity measures such as pay by- the -load or mile may encourage excessive driving speed, increased stress and working while fatigued to meet deadlines and financial pressures. Suggestions by drivers for training and education within truck driving school curriculum at the start of a driving career as well as ongoing educational strategies tailored to transport drivers such as mentoring by experienced drivers and coaching new hires were suggested as ways to help create a realistic picture of the driver’s world and enhance recruitment of younger drivers into the profession. Drivers see other drivers as “family” on the road, and see themselves as being very independent. As one driver commented “…we are set in our ways because you work on your own…no one is looking over your shoulder”.

Drivers commented that attitudes of their colleagues in the sector also need to change. They recognized an attitude of helplessness and inaction among their profession regarding health as exemplified by this comment “there are a lot of old school drivers out there and it’s become accepted that if you’re a truck driver you’re not going to be in the best of health. It’s just been that way for years and a lot of guys just come to accept it. …. And then you get so far into a situation where your knees are shot, your back shot, a 50 pound gut hanging around the middle of you and now where and how are you going to start exercising”.

#### Family

Missing family commitments due to the nature of the work (the long hours, last minute load demands by customers) was mentioned by several of the drivers, but this driver stressor was not mentioned by the managers. Drivers regularly commented on the importance of family time to them, which they felt should be more valued and recognized by the company.

#### Health conditions and access to care

Health conditions and risk factors such as smoking, weight concerns, high blood pressure and diabetes were mentioned by both drivers and managers as important health and economic issues. Suggestions were made by both drivers and managers to enhance access to programming to increase awareness for preventive action. However, it was unclear where, what and how that programming would take place and who would provide it. Having access to primary health care options at truck stops would fit with the independent driver penchant for self-management (e.g. blood pressure machines to monitor their own blood pressures and keep records for their health care provider).

Lack of access to basic and preventive health care due to scheduling issues and long unpredictable hours was also mentioned by most of the drivers. Drivers discussed going to walk- in clinics, not their regular primary care provider. They also described trouble keeping scheduled appointments, which resulted in being dropped as patients.

### Fatigue

Although hours of service regulations are in place, long workdays are permitted. Many of the drivers reported that they spent on average 14–16 hours a day on duty with up to70 total driving hours allowed by regulation in an 8- day schedule. Drivers noted that after a 10 to 14 hour day they were exhausted and unable to properly participate in extra health promoting activities. Many independent drivers however commented that the regulation of capping hours of driving to 70 hours in an 8 day schedule has affected their take home income, making them more financially stressed. They felt that they as drivers understood their sleep needs, were adjusted to less sleep and should be allowed to set their own sleep patterns without regulation.

Managers recognized that the drivers’ jobs are not normal jobs as they work ‘on demand’ with irregular hours. The job is not like an office job with clear time frames and schedules. Mental fatigue of drivers was accepted as part of the job. Drivers identified various techniques to keep alert: rolling the window down for fresh air, smoking more, drinking coffee or 5- hour energy drinks, walking around the truck, and taking 5 minute naps to avoid dangerous drowsiness while driving. Managers emphasized adherence to the regulations due to traffic accident issues that can undermine the company safety rating and thus a company’s competitiveness.

## Discussion

Health concerns of truck drivers in this study related to stress, diet, exercise and fatigue. These health risks were discussed as relating directly to the job demands in terms of long unpredictable work hours, financial pressure and the sedentary nature of the job. Both transport truck drivers and managers were aware of the profession’s potential to foster lifestyle related chronic diseases but perceived substantial challenges in making the profession more amenable to promoting healthy lifestyles. There was general agreement that health interventions need to be effective and the approach must be cost-effective and tailored to the irregular work schedules.

Many of the findings were consistent with studies conducted in the United States and internationally documenting lifestyle risks among truck drivers especially for cardiovascular disease [19-28]. However, substance use and risky sexual behaviour, which have been identified in other studies, were not mentioned in the discussions. We did not specifically ask about these issues for concern that it could have limited the willingness of drivers to participate. Cardiovascular disease related risks were the dominant lifestyle issues discussed.

The findings from this study add to our understanding of the occupation related health challenges of lone remote workers; and in this case workers who are mostly male. This study provides the basis for future work to improve health outcomes of the transport driver population and to examine workplace health issues from a gender analysis male perspective. Through the partnerships with the trucking companies developed during this study, there may be increased organizational capacity to assist with the movement, transfer, and flow of this new knowledge through the industry.

However, there are several limitations to this study. The specific business focuses and workplaces of the participating companies may not represent other companies across Canada, thereby limiting generalizability of participant’s attitudes regarding occupational issues to other companies. Most of the focus group participants were short-haul drivers; therefore the lifestyle issues of long-haul drivers who are away from home for days to weeks, were less represented. Most of the drivers were also company drivers with benefits and secure, stable pay. Independent contracted drivers, who identified an added stress of financial insecurity, were less represented.

The FG format might have hindered sharing of differing views and personal experiences among the group. In comparison to drivers, the smaller FG sizes with managers appeared to elicit different perspectives and a more critical review of environmental factors. Holding the FGs at the trucking company’s office was helpful for convenience; however, it might have limited participants’ sharing, even though confidentiality of the discussions was stressed.

## Conclusions

Although truck drivers and managers identified health conditions and concerns related to the transportation sector occupation, they further indicated that their working conditions exacerbated these issues because of the complex, competitive working environment. Planning for and incorporating health and wellness strategies into the for –profit trucking operations and company corporate policies becomes a challenge. Truck drivers are ‘lone workers’ - their workplace is their cab, the road and the stops along the journey. This also complicates developing traditional workplace health interventions to address a healthy lifestyle.

The themes from this qualitative study relating to health and lifestyle provided relevant areas of inquiry to develop and implement a health and wellness survey implemented in 13 companies in Southern Ontario in order to provide robust evidence of health and lifestyle issues relating to the occupation and to assist in the development of effective strategies to mitigate health risks [28,29]. Standard questionnaires and tools relating to the themes were identified from the literature and used where possible [29]. The next steps will be to work with companies, drivers and other stakeholders to develop mechanisms to identify and evaluate how drivers wish to receive information, the environmental supports and policies necessary to make healthy choices, and to create, implement and evaluate tailored, targeted, cost effective health promotion messages, policies and uptake strategies. The findings from this study will provide an understanding of the issues from the drivers and managers viewpoints to ensure that the knowledge and support tools created are tailored towards a non traditional workplace that poses challenges to healthy lifestyles.

## Availability of supporting data

The data set supporting the results of this article is included within the article and its additional files.

## Competing interests

The authors declare that they have no competing interest.

## Authors’ contributions

BM and MH conceived the study. BM, MH and FML were responsible for developing the qualitative focus group questions and assisted in collection of data. BM, MH, RA, JR and FML analysed the data. BM and SL wrote the first draft of the manuscript. BM, MH, RA, JR, LD, FML contributed to the interpretation of the results, commented on drafts and approved the final version of the manuscript. All authors read and approved the final manuscript.
